# Development and validation of a high performance liquid chromatography method to determine nevirapine in plasma in a resource-limited setting

**DOI:** 10.4102/ajlm.v8i1.880

**Published:** 2019-05-16

**Authors:** Faithful Makita-Chingombe, Andrew J. Ocque, Robin DiFrancesco, Charles Maponga, Farai Muzambi, Tsitsi G. Monera-Penduka, Tinashe Mudzviti, Takudzwa J. Mtisi, Gene D. Morse

**Affiliations:** 1 International Pharmacology Specialty Laboratory, School of Pharmacy, University of Zimbabwe College of Health Science, Harare, Zimbabwe; 2 Center for Integrated Global Biomedical Sciences, School of Pharmacy and Pharmaceutical Sciences, University at Buffalo, Translational Pharmacology Research Core, New York State Center of Excellence in Bioinformatics and Life Sciences, The State University of New York, Buffalo, New York, United States

**Keywords:** high performance liquid chromatography, nevirapine determination, method development and validation

## Abstract

**Background:**

There are several instances where nevirapine pharmacokinetic monitoring may be useful, such as in special populations or pharmacokinetic drug interaction studies that require the ascertainment of nevirapine pharmacokinetics in the sub-Saharan region.

**Objectives:**

The main aim of this study was to produce a validated, sustainable and relevant nevirapine assay method that meets bio-analytical regulatory requirements.

**Methods:**

The developed method utilised a Waters 2795 Alliance high performance liquid chromatography system with a 2996 photo diode array detector, an Atlantis dC18 5 micron, 3.9 mm × 150 mm analytical column and a gradient flow rate of 1 mL/min. Ultraviolet detection data were collected from 210 nm to 400 nm, extracted at 260 nm, and processed for nevirapine and internal standard peak height responses.

**Results:**

The method proved to be linear (R2 0.995), precise (+1.92% – +9.69%) and accurate (-9.70% – 12.0%). Recovery for the analyte and internal standard was between 98.8% and 114%. The method showed good specificity as no interferences were caused by common African traditional medicines, anti-tuberculosis medications or other concomitant antiretrovirals nor endogenous components.

**Conclusion:**

The method is reproducible, relevant to our setting and uses considerably low plasma volumes with preservation of some consumables, a desirable key factor in a resource-limited setting.

## Introduction

While nevirapine is no longer the preferred first-line non-nucleoside reverse transcriptase inhibitor for the management of HIV, it is still relevant as an option in Zimbabwe. Nevirapine is also available for use in HIV-exposed neonates for the prevention of mother-to-child transmission.^1^ In this regard, a validated method to determine nevirapine concentrations in plasma is desirable. Research initiatives supported by several United States National Institutes of Health programmes, experienced mentors and commercial donations have contributed to the establishment of a clinical pharmacology laboratory to support HIV research in Zimbabwe.^2^ However, method development and validation present resource and infrastructural challenges in resource-limited settings (RLS).

Many limitations need to be considered during method development. The cost of equipment and availability of reagents and supplies are major determinants affecting the feasibility and sustainability of the method. Patient factors such as concomitant medications and blood testing volumes must also be considered to assure method sensitivity and specificity challenges. There is evidence indicating that in African settings, people on antiretrovirals also utilise traditional herbal medication.^3^ High performance liquid chromatography (HPLC) assays become compromised with introduction of diverse excipients, metabolites or active compounds eluting at the same retention time and absorbing at the same wavelength as the analyte.^4^ Therefore, assessing for interference of concomitant antiretrovirals, traditional herbal medicines and medications for potential opportunistic diseases, such as anti-tuberculosis drugs, is relevant when developing assay specificity in RLS. The sample matrix itself should also have minimal method interference. Due to the need for frequent monitoring, HIV-positive patients will also have several tests that require drawing of blood.^5^ Therefore, to minimise blood collection volumes, a sensitive assay method accommodating small sample volumes should be pursued.

When preparing a sample for assay, extraction and concentrating steps can be optimised to improve method sensitivity. Different techniques for extraction of analyte from the plasma matrix are employed. These vary from an expensive solid phase extraction (SPE) such as cartridge extraction to a less expensive extraction approach such as protein precipitation using solvents. Furthermore, a cost-effective but efficient sample concentration technique is an important factor. Techniques to concentrate samples vary from nitrogen-dependent evaporation to centrifugal force applications. Nitrogen evaporation is employed in some methods.^6,7^ However, this has limited application in RLS due to the difficulty in accessing nitrogen gas. In addition, in RLS reagents and materials are usually expensive due to the need for importation, which contributes to increased assay costs. Sustainability of an assay also depends on the stability of these reagents.

Mass spectrometry-based methods have been employed in plasma assays for antiretrovirals in several pharmacology and pharmaceutical laboratories.^8,9^ These assays offer greater sensitivity and specificity when compared to ultraviolet-based detection.^10,11,12^ However, mass spectrometry-based analytical systems are expensive to acquire and maintain.^13^ Furthermore, to avoid matrix effects frequently encountered with clinical plasma samples, costly internal standards are required. HPLC with ultraviolet detection becomes a viable option when considering an assay for nevirapine in patients taking nevirapine as prescribed, because the plasma concentrations are fairly high and detectable by ultraviolet-based methods.^14^

Despite limited resources, methods developed should still abide by regulations on method validation and be as effective (e.g., appropriate range of quantitation and specificity) as assaying the same analyte by mass spectrometry. Bio-analytical method guidelines issued by national agencies accepting drug submissions as well as medical laboratory accreditation agencies provide key support for assuring laboratory performance measures. Therefore, developing the capacity to monitor antiretroviral concentrations within local research institutions requires striking a balance between cost effectiveness and development of a robust method that ultimately does not compromise the integrity of sample results.^10^

The aim of this study was to develop and validate a suitable HPLC method, using ultraviolet detection to determine nevirapine concentrations in human plasma in Harare, Zimbabwe, a resource-limited setting. The method was required to meet the standard requirements for sample analysis used to support clinical trials that incorporate nevirapine plasma concentration as an outcome measure.^15^

## Methods

### Ethical considerations

The method application was done using samples collected in 2013 at Parirenyatwa HIV Opportunistic Infections Clinic, Harare, Zimbabwe for a project trial approved by the Joint Research Ethical Committee 130/10 in Harare, Zimbabwe, and the Medical Research Council of Zimbabwe /B/255 in Harare, Zimbabwe (registration number NCT01410058).

### Method development

#### Chemicals, reagents, and equipment

All antiretrovirals used (nevirapine, indinavir, efavirenz, atazanavir, ritonavir, lopinavir, zalcitabine, lamivudine, tenofovir, zidovudine, stavudine, abacavir and emtricitabine) were obtained from the National Institutes of Health AIDS Research and Reference Reagent programme. Nevirapine powder had a stated purity of 90% and indinavir powder, which was used as the internal standard (IS), had a stated purity of 89%. Human, heparinized plasma was donated by the National Blood Transfusion Services (Harare, Zimbabwe). HPLC-grade methanol and acetonitrile were purchased from Microsep, ROMIL Pure Chemistry (Johannesburg, South Africa). Reverse osmosis water was produced from an ELGA PURELAB^®^ water purifier (Harare, Zimbabwe). The HPLC analyses were performed using a Waters 2795 separation module (Waters Technologies Corporation, Milford, Massachusetts, United States) equipped with Waters 2998 PDA detector and Empower software (version 3; Waters Associates, Milford, Massachusetts, United States).

#### Instrumental and analytical conditions

HPLC, as described above, employing a Waters Atlantis dC18 3.9 mm × 20 mm, 5 *µ*m preceded by a guard column was used. The final mobile phase consisted of (1) 10 mm ammonium acetate, acetonitrile and methanol (60:25:15) pH4 and mobile phase (2) 10 mm ammonium acetate, acetonitrile and methanol (20:50:30) pH4 and was delivered using the following gradient scheme: 0–3 min (100% 1), 4 min – 7 min (100% 2), and 7.01 min – 10 min (100% 1). The flow rate was 1 mL/min. The auto-sampler was set at ambient temperature and the column oven was maintained at 40 °C. The injection volume of sample was 65 *µ*L. Scanning was from 210 nm to 400 nm with data extracted at 260 nm for nevirapine and indinavir using Empower Software Version 3. These conditions were used for method validation carried out based on the United States Food and Drug Administration guidelines.^15^

#### Sample treatment

Five mg of nevirapine and IS reference grade powders were individually weighed on a calibrated analytical balance. Both drugs were separately dissolved in 100% methanol and dilutions for their working standards were done in 50:50 methanol:water. IS was spiked into calibration standards, quality controls or unknowns prior to extracting nevirapine by protein precipitation using cold acetonitrile. Initial testing of plasma sample amounts ranged from 100 *µ*L to 250 *µ*L with various adjustments of precipitation solution. Samples were then vortexed and centrifuged for 10 min at 7852 × g. A volume of the supernatant was transferred to a culture tube and evaporated in a CentriVap^TM^ (VWR Labconco, Kansas City, Missouri, United States) benchtop centrifugal vacuum evaporator at 50 °C until dry. The dried sample was reconstituted with 200 *µ*L mobile phase before being transferred to a polypropylene insert and placed in an auto-sampler vial. The inserts and vial tops were discarded after use while the vials were preserved for reuse.

#### Chromatography

Using various columns and different mobile phase conditions, optimisation of chromatographic separation of nevirapine and IS was performed. Sample carryover was ruled out by injecting mobile phase after injections of prepared plasma samples at the following nevirapine concentrations: 500 ng/mL, 1000 ng/mL, 5000 ng/mL and 10 000 ng/mL. Mobile phase chromatograms of several sources of nevirapine-free plasma samples were inspected to ensure absence of peaks within the retention time window of both nevirapine and IS. A concentration of 500 ng/mL was indicated as the lowest calibrator value with analyte response greater than 20% of the blank response. Consequently, the lowest calibrator was the limit of quantification (LOQ) as per FDA guidance. Repeatability assessments were done at LOQ by carrying out six injections at an injection volume of 65 *µ*L.

### Method validation

#### Calibration curves and quality control preparation

Calibration concentrations were prepared from nevirapine stocks weighed separately from the stock used for quality controls (QC). Calibration concentrations of 15 000 ng/mL, 13 000 ng/mL, 8000 ng/mL, 4000 ng/mL, 2000 ng/mL, 1000 ng/mL and 500 ng/mL were prepared based on the therapeutic range for nevirapine in plasma.^16,17,18^ The lower LOQ (LLOQ) was prepared at 500 ng/mL, lower quality control (LQC) at 1500 ng/mL, middle quality control (MQC) at 5000 ng/mL and highest quality control (HQC) at 12 000 ng/mL by dilution of stock solution into blank human plasma.

#### Intra and inter-day precision and accuracy of calibration curve, lower limit of quantification and quality controls

A calibration curve was constructed for measuring every batch of validation samples with 1/x^2^ weighted linear regression. Six samples were tested at each QC level including LLOQ on 3 days; a different analyst performed the analysis each day. Accuracy and precision of dilution was measured using a prepared high out of quantitation quality control (HOQ) at 45 000 ng/mL. Dilution of the HOQ was carried out in two different dilutions, 1:4 and 1:8 using blank plasma as diluent.

#### Stability

Freeze-thaw, reinjection and room temperature stability were determined at low and high QC concentrations in triplicate and compared to freshly prepared controls stored at (-70 °C). Room temperature stability involved leaving the samples on the bench top for 6 h. Freeze-thaw was performed over three freeze (-70 °C) and thaw (room temperatures) cycles. Reinjection stability samples were assessed by re-injecting samples stored at 4 °C for 168 h.

#### Specificity and selectivity

Selectivity and specificity were investigated by assessing six different blank plasma samples and monitoring for appearance and potential interference by endogenous compounds. Interference from other antiretrovirals (stavudine, tenofovir, abacavir, lamivudine, zidovudine, emtricitabine, atazanavir, ritonavir, lopinavir, efavirenz, dideoxycytidine), anti-tuberculosis drugs (isoniazid, rifapentine, pyrazinamide, desacetylrifapentine) and herbal supplements *Allium sativum* (garlic) and *Moringa oleifera* Lam. were tested by spiking each component separately in blank plasma. Twenty *µ*L of 10 *µ*g/mL for antiretrovirals and 50 *µ*g/mL for anti-tuberculosis drugs were spiked into 180 *µ*L of plasma and processed as described in the method. One whole *Syzygium aromaticum* (clove) and one gram each of *Allium sativum* (garlic) and *Moringa oleifera* Lam. were ground before extraction in methanol. After centrifugation for 15 min, 20 *µ*L of the extract supernatant was spiked into 180 *µ*L plasma before the sample was processed. Individual component chromatograms were overlaid with LLOQ chromatograms.

#### Recovery

Recovery was performed at the HQC and LQC levels in six independent lots of human EDTA plasma (tests) and compared to the response observed in water (controls). To establish a control, plasma was replaced with water and the sample treated as described.

#### Method application

The method has been routinely used in proficiency testing in an external quality assurance programme conducted by the United States National Institute of Allergies and Infectious Disease and the Division of AIDS Clinical Pharmacology Quality Assurance programme, which conducts proficiency testing assessments for clinical pharmacology laboratories under the National Institutes of Health HIV research network.^19^ Methodology utility was demonstrated by assessing reproducibility within ±20% by re-analysis of four patient samples from a previously reported investigation on effect of *Moringa oleifera* Lam. leaf powder on the pharmacokinetics of nevirapine in HIV‑positive adults.^20^ Patients were on nevirapine 200 mg twice daily and samples were taken at timed intervals after consumption of *Moringa oleifera* Lam. (1.85 g). The trial registration number is NCT01410058, JREC 130/10, MRCZ/B/255.

## Results

### Method development

Sample treatment was successful using protein precipitation (400 *µ*L acetonitrile), and a minimal amount of plasma sample (180 *µ*L) was sufficient for the method. Centrifugal vacuum evaporation, which improved sensitivity, was successfully employed using 550 *µ*L of the sample supernatant (85% volume) using the CentriVap^TM^. Using auto-sampler vial inserts preserved the containment vials for reuse thus lowering consumable costs.

**FIGURE 1:**
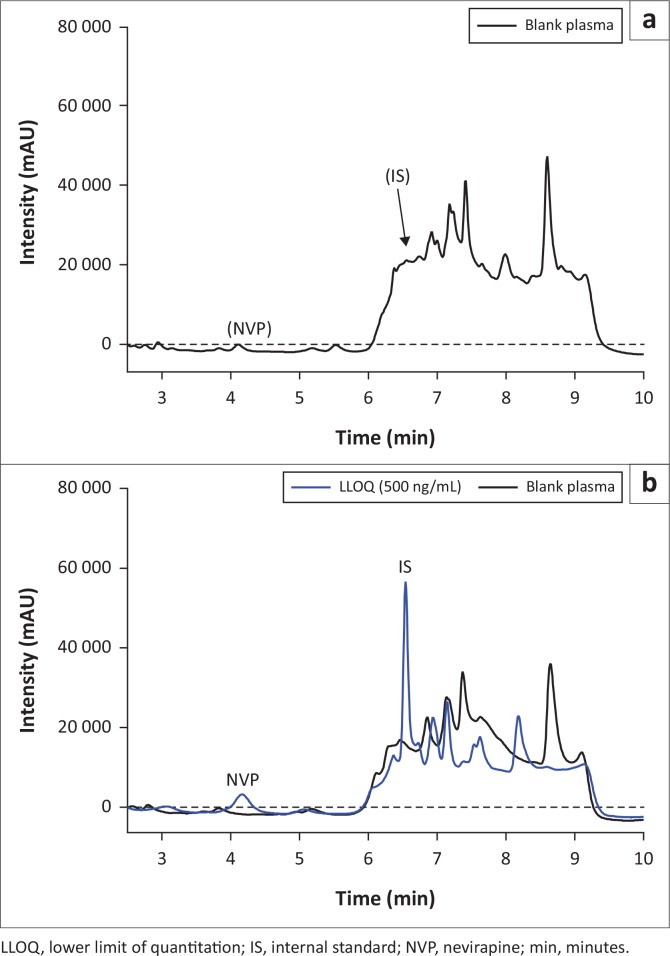
Chromatograms of blank plasma (a) and blank plasma spiked with internal standard and nevirapine (b) showing no endogenous components interference at nevirapine and internal standard retention times.

Gradient conditions at pH4 were ideal in improving sensitivity and selectivity. Desirable retention times of 4.5 min for nevirapine and 5.9 min for indinavir were achieved within a run time of 10 min. Chromatographic separation of nevirapine and IS from each other and from endogenous plasma components was shown by an overlay of blank and LLOQ chromatograms (Figure 1). Absence of sample carry over and high repeatability (5.39%) indicated that the method was ready to move to the validation phase.

### Method validation

**FIGURE 2:**
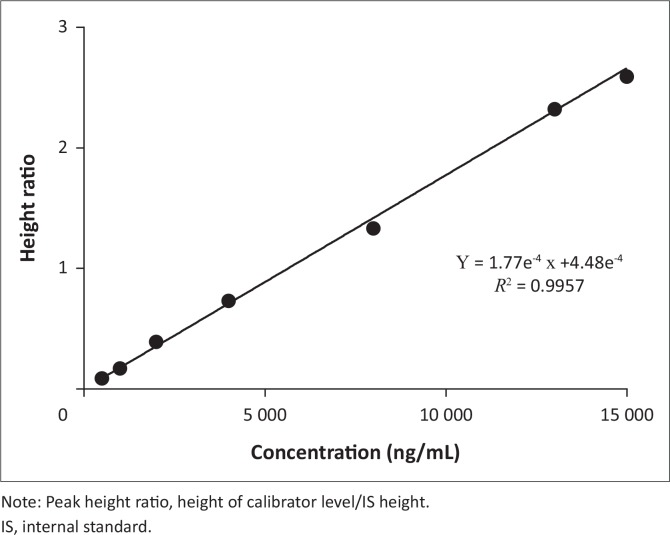
Representative nevirapine calibration curve for inter-day precision and accuracy.

The calibration curve was linear over the calibration range of 500 ng/mL – 15 000 ng/mL. An example of a calibration curve obtained for nevirapine is illustrated in Figure 2. Where a calibrator level other than the LLOQ was not within ±15%, the calibrator was omitted and the curve recalculated. The accuracy range was between −8.13 and +8.83% with a precision of < 12%. Precision and accuracy of the QC samples were within acceptance criteria of United States Food and Drug Administration and are summarised in Table 1. Precision was < 9.69% and accuracy ranged from −9.70 to +12.0% for LQC, MQC and HQC. LLOQ precision was < 7.53% and accuracy was +2.76% to +16.1%. HOQ precision was < 2.84% and accuracy was +4.96% to +5.15% whether diluted 1:4 or 1:8.

**TABLE 1:** Inter and intra day accuracy and precision of quality control samples at University of Zimbabwe International Pharmacology laboratory, 2016.

QC	Target (ng/mL)	Intra-day 1				Intra-day 2				Intra-day 3				Inter-day			
		Mean ± SD	CV%	Accuracy%	*n*	Mean ± SD	CV%	Accuracy%	*n*	Mean ± SD	CV%	Accuracy%	*n*	Mean ± SD	CV%	Accuracy%	*n*
LLOQ	500	527 ± 35	6.59	5.30	6	580 ± 13.6	2.35	16.1	6	514 ± 37	7.19	2.76	5†	542 ± 40	7.53	8.35	17
LQC	1500	1551 ± 29	1.92	3.39	6	1511 ± 123	8.14	0.78	6	1355 ± 58	4.29	-9.70	6	1395 ± 115	8.28	-7.01	18
MQC	5000	5342 ± 277	5.18	6.83	6	5529 ± 429	7.75	10.6	5‡	4616 ± 199	4.32	-7.70	6	5141 ± 498	9.69	2.81	17
HQC	12 000	12 562 ± 521	4.15	4.68	6	13 434 ± 661	4.92	12.0	6	11 339 ± 486	4.28	-5.51	6	12 445 ± 1030	8.27	3.71	18
HOQ (1:4)	45 000	47 232 ± 1334	2.82	4.96	3	-	-	-	-	-	-	-	-	-	-	-	-
HOQ (1:8)	45 000	47 317 ± 1346	2.84	5.15	3	-	-	-	-	-	-	-	-	-	-	-	-

QC, quality controls; LQC, lower quality control; LLOQ, lower limit of quantitation; MQC, middle quality control; HQC, highest quality control; IS, internal standard; HOQ, quantitation quality control; SD, standard deviation; n, number; CV, coefficient of variation (precision).

† , LLOQ had double IS.

‡ , MQC had no IS, values were excluded.

**FIGURE 3:**
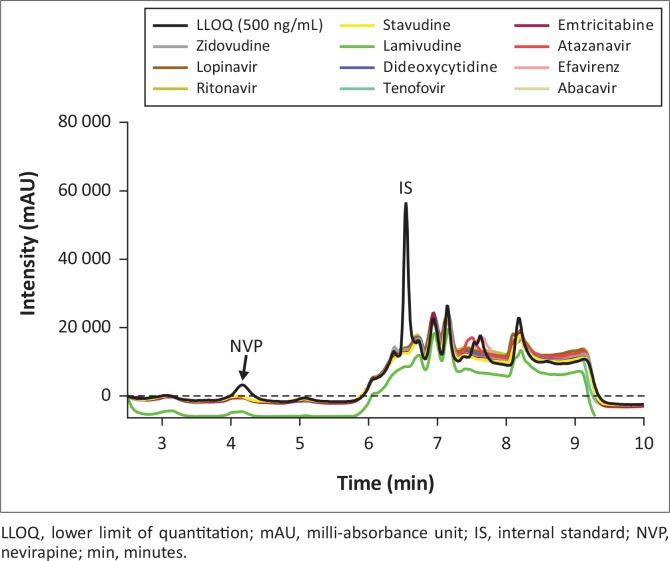
Chromatogram overlay of LLOQ with antiretrovirals, no interference was observed at nevirapine and internal standard retention times for all antiretrovirals.

**FIGURE 4:**
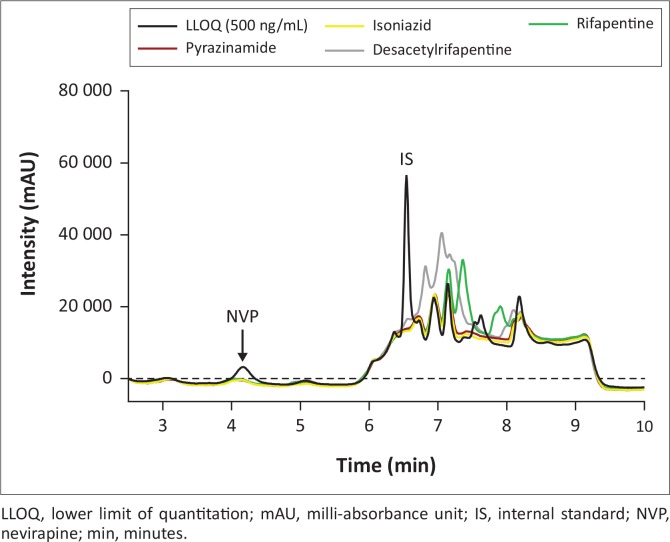
Chromatogram overlay of LLOQ with anti-tuberculosis drugs; no interference was observed at nevirapine and internal standard retention times.

**FIGURE 5:**
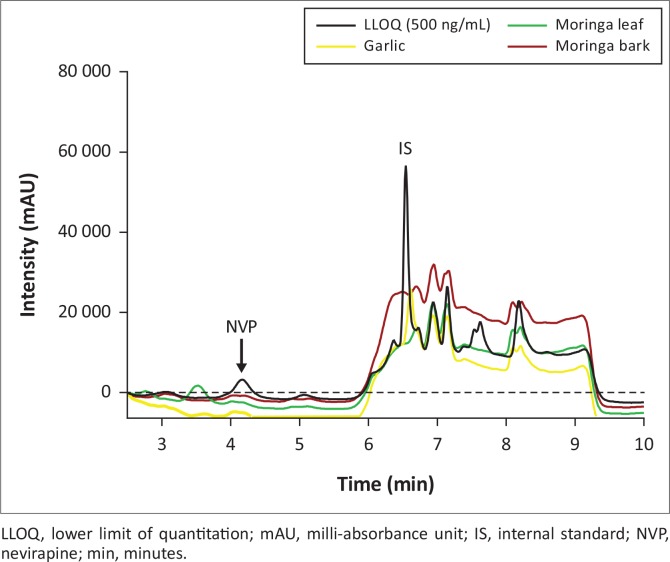
Chromatogram overlay of LLOQ with herbs, no interference at nevirapine and internal standard retention times was observed in all herbs.

The deviations from nominal concentrations for freeze-thaw, room temperature and reinjection stability were also within acceptable limits (Table 2). None of the concomitant medications (Figures 3, 4 and 5) interfered with the analyte or IS, including additional antiretrovirals other than those taken in combination with nevirapine. Achieved recovery for the analyte ranged from 103% to 114%, while the indinavir IS recovery ranged from 98.8% to 113%.

**TABLE 2:** Nevirapine stability over different conditions at University of Zimbabwe International Pharmacology laboratory.

Variable	QC	Target (ng/mL)	Freeze-Thaw			Room temperature			Reinjection		
			Mean ± SD	% bias	% deviation	Mean ± SD	% bias	% deviation	Mean ± SD	% bias	% deviation
Controls	LQC	1500	1466 ± 48.8	-2.26	-	1450 ± 59.9	-3.33	-	1340 ± 72.0	-10.7	-
	HQC	12 000	11 738 ± 165	-2.18	-	11 761 ± 611	-1.99	-	11 417 ± 739	-4.86	-
Treated	LQC	1500	1508 ± 64.6	0.533	2.86	1528 ± 68.4	1.87	5.38	1462 ± 16.1	-2.53	9.10
	HQC	12 000	11 580 ± 220	-3.50	-1.34	12 497 ± 372	4.14	6.26	12 338 ± 913	2.82	8.07

Note: % bias is the comparison of the measured value to the target value, % deviation is the comparison of the treated value to the control values.

*N* = 3.

LQC, lower quality control; HQC, highest quality control; SD, standard deviation.

### Method application

**FIGURE 6:**
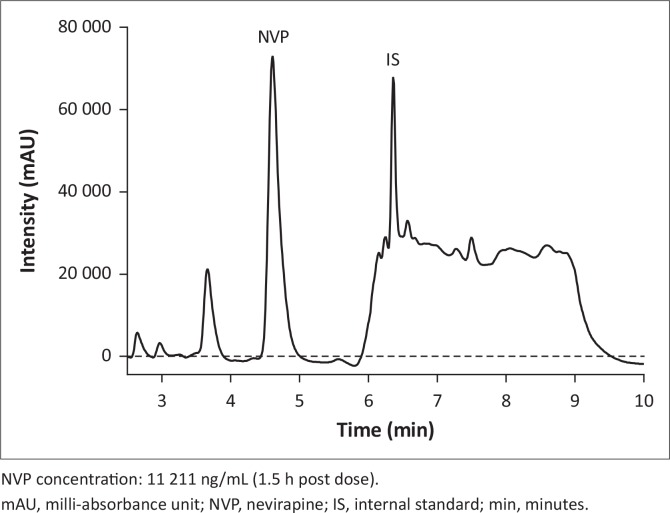
Chromatogram of patient sample. (Samples collected at Parirenyatwa Opportunistic infections clinic in 2013 and assayed at University of Zimbabwe International Pharmacology laboratory).

All samples analysed in the Clinical Pharmacology Quality Assurance programme external proficiency tests using this validated method were acceptable (accuracy from target values within 20%). The percentage bias for this method from the previously assayed patient samples was within 20% of reported values (-13% to +17% difference); an example of a patient chromatogram is shown in Figure 6.

## Discussion

An optimised HPLC-ultraviolet-based method for nevirapine determination in plasma was successfully developed and validated within a resource-limited setting. Optimisations in assay development aimed at attaining a desirable chromatogram, ultraviolet detection wavelength, resolution and retention times for both the analyte and IS in human plasma matrix. A minimal amount of 180 *µ*L plasma was sufficient compared to other HPLC-based nevirapine assay methods that utilised 500 *µ*L or more.^21,22,23^ Low sample amounts are desirable due to the need for frequent monitoring of HIV patients^4^ and the need for additional laboratory tests during antiretroviral treatment. While most sample treatment methods use SPE cartridges, protein precipitation has been used with success by other researchers.^24,25^ Even though SPE may result in a cleaner sample, the technique requires consumable SPE products and supporting interfaces such as manifolds and vacuum pumps that come at a greater cost than this method. Employing centrifugal vacuum evaporation resulted in improved sensitivity and excluded the use of nitrogen gas, which is not locally produced. To reduce column burden and sustain longer column life, an inexpensive guard column was placed in line prior to the column. In addition, the method is designed to increase organic components in the gradient, helping to remove endogenous components and debris from the column. The analysis run time was shorter than that observed in other gradient or isocratic HPLC-based nevirapine assay methods.^21,22,23^ The pH controlled mobile phase composition of the gradient method capitalised on the high solubility of nevirapine in organic solvents to achieve an early elution of nevirapine.^26^ The observed nevirapine and indinavir stability under different environmental subjections was reflective of analyte and IS stability in plasma as proved by other scholars.^27^ To assure specificity of the method, several regional considerations were: national formularies for co-administered HIV antiretrovirals, as well as treatments for common co-infections and the local use of herbal supplements particularly *Moringa oleifera* Lam. and *Allium sativum* (garlic).^3^ In that regard, and as achieved, other antiretrovirals, anti-tuberculosis drugs and indicated herbs should not interfere with or compromise the assay method. Observed recoveries were comparable to those achieved by other extraction methods, for example by SPE.^28,29^ The results during method application further confirmed the method’s specificity.

### Conclusion

A valid method to measure nevirapine in plasma was developed by using HPLC-ultraviolet detection and resource-conserving techniques while maintaining the sensitivity, specificity, selectivity, accuracy and precision needed to monitor nevirapine at therapeutic ranges. The described method is evidence that despite limited resources, capitalisation of viable resources enables establishment of effective drug analysis methods relevant to RLS.
